# Gigantic retroperitoneal hematoma as a complication of anticoagulation therapy with heparin in therapeutic doses: a case report

**DOI:** 10.1186/1752-1947-2-162

**Published:** 2008-05-17

**Authors:** Stavros I Daliakopoulos, Andreas Bairaktaris, Dimitrios Papadimitriou, Perikles Pappas

**Affiliations:** 1 Herz- und Diabeteszentrum Nordrhein, Westfalen, Georgstrasse, Bad Oeynhausen, Universitätsklinikum der Ruhr-Universität Bochum, Germany; 2 Department of Vascular and Endovascular Surgery, 424 Military Hospital, Thessaloniki, Greece

## Abstract

**Introduction:**

Spontaneous retroperitoneal hemorrhage is a distinct clinical entity that can present as a rare life-threatening event characterized by sudden onset of bleeding into the retroperitoneal space, occurring in association with bleeding disorders, intratumoral bleeding, or ruptures of any retroperitoneal organ or aneurysm. The spontaneous form is the most infrequent retroperitoneal hemorrhage, causing significant morbidity and representing a diagnostic challenge.

**Case presentation:**

We report the case of a patient with coronary artery disease who presented with transient ischemic attack, in whom anticoagulant therapy with heparin precipitated a massive spontaneous atraumatic retroperitoneal hemorrhage (with international normalized ratio 2.4), which was treated conservatively.

**Conclusion:**

Delay in diagnosis is potentially fatal and high clinical suspicion remains crucial. Finally, it is a matter of controversy whether retroperitoneal hematomas should be surgically evacuated or conservatively treated and the final decision should be made after taking into consideration patient's general condition and the possibility of permanent femoral or sciatic neuropathy due to compression syndrome.

## Introduction

Hemorrhage is the most important complication of unfractionated heparin in patients with atrial fibrillation (AF) treated with oral vitamin K antagonist (VKA) during hospitalization or among those receiving anticoagulants in terms of emergency or elective cardiac surgery [1,2] or in the initial treatment of deep venous thrombosis [3,4].

Analysis of the data presented by the European AF Trial Study Group [5] shows that as the international normalized ratio (INR) increased, there was an increase in the risk of major bleeding, such that at INR ≥ 5.0, the risk of bleeding increased 3.6-fold relative to INR ≤ 2. The optimal intensity of anticoagulation that achieved maximum therapeutic effect with minimum risk was determined to be at INR = 3.0. These data, along with recommendations from the recent American College of Chest Physicians (ACCP) guidelines, indicate that the optimal intensity of anticoagulation for balancing efficacy in presenting stoke, while minimizing the risk of bleeding, is within the range INR = 2.0–3.0 (see [6]). Among outpatients receiving oral anticoagulants those with INR ≥ 6.0 face a significant risk of major hemorrhage [7].

Retroperitoneal hemorrhage is most frequently seen after femoral artery catheterization or pelvic and lumbar trauma [8-10]. In the absence of trauma, retroperitoneal hemorrhage most frequently results from a ruptured abdominal aortic aneurysm or bleeding from an underlying condition in the kidneys or adrenal glands. Spontaneous retroperitoneal hemorrhage (SRH) denotes bleeding without any known inciting trauma or underlying retroperitoneal pathology. SRH is uncommon and is almost exclusively seen in association with anticoagulation states, coagulopathies and hemodialysis [11,12].

A plethora of conditions have been used as a possible hypothesis of the pathophysiology of SRH. Unrecognized minor trauma in the microcirculation in the presence of coagulopathy has been suggested [13,14].

The surgeons' quiver contains various types of approach to the treatment of this relatively uncommon complication such as conservative management, angiographic evaluation, percutaneous embolization or surgical intervention.

## Case presentation

A 57-year-old Caucasian male was admitted to our hospital presenting with focal ischemic cerebral neurological deficit of acute onset. The patient had had an acute non-Q-wave myocardial infarction episode 11 years ago and post-infarct had undergone percutaneous transluminal coronary angioplasty: ramus circumflexus in 1995 and ramus diagonalis I in 1997. Eleven months before admission, an evaluation elsewhere had revealed persistent atrial fibrillation and since this evaluation the patient had been receiving Warfarin and had maintained INR = 2.0–2.5.

On the day of admission, examination of the patient revealed intense dizziness with diplopia, instability and complete left-sided homonymous hemianopsia. The findings suggested a transient ischemic attack involving the anterior circulation: carotid artery territory.

There was no personal or family history of coagulopathy or stroke, valvular heart disease trauma, chest pain or illicit intravenous drug usage. He smoked 20 cigarettes daily and consumed alcohol in moderation in the past.

The prothrombin was normal, INR = 2.4, the partial thromboplastin time was 45 seconds, the values for urea, nitrogen, creatinine, glucose, uric acid, bilirubin, phosphorus, electrolytes, creatinine kinase, lactate dehydrogenase, amylase and alkaline phosphatase were normal. An electrocardiogram (ECG) revealed atrial fibrillation at a rate of 110, with nonspecific ST-segment and T-wave abnormalities. A radiograph of the chest showed clear lungs and slight cardiac enlargement. A cardiac ultrasonographic examination showed no vegetations, intracardiac thrombus, segmental wall-motion abnormalities or intracardiac shunts. A test for the erythrocyte sedimentation rate was normal, as were tests for antinuclear antibodies, lupus anticoagulant and antiphospholipid antibody.

Computed tomography (CT) brain imaging was performed without the use of contrast material, but failed to indicate hemorrhage, infarct, abscess, tumor or cerebral metastasis. Heparin 20,000IE/24 hours intravenously and Metoprolol 100 mg by mouth were administered. Repeated physical examinations and ECGs showed no changes.

On the second hospital day the patient awoke with a slight neurologic deficit that gradually progressed in a stepwise fashion. Hemiplegia (upper left extremity and face were involved), hemianesthesia and Babinski sign contralateral to the hemiparesis were established. He had mild dysarthria, but his speech was fluent and his comprehension, repetition and naming abilities were intact. CT brain imaging was performed and no hemorrhagic transformation was found. Dipyridamole 200 mg/day, Aspirin 25 mg/day, Heparin 20,000IE/24 hours and Mannitol 20% solution (1 g/kg) were administered. Daily monitoring of ECG, vital signs, electrolytes, blood urea nitrogen, creatinine, urine output showed no changes.

On the fifth hospital day the patient noted the acute onset of pain in the lower right abdominal quadrant and lumbar region accompanied by mild nausea. The patient held the right hip in flexion and external rotation. Any attempt to straighten the leg aggravated the pain with radiation to the medial and anterior portions of the lower extremity Weakness of the right quadriceps femoris muscle, paresthesia over the anterior thigh and right flank were evident. The partial thromboplastin time was 43 seconds and INR = 2.4.

**Figure 1:**
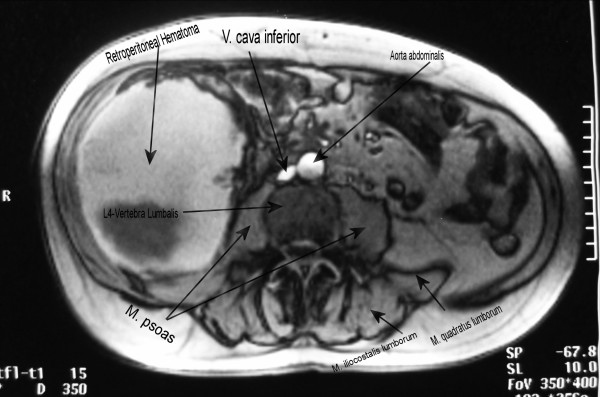
MRI – transverse plan (L4) with IV contrast gadolinium-BOPTA, revealing a well-defined mass, a huge retroperitoneal hematoma.

**Figure 2:**
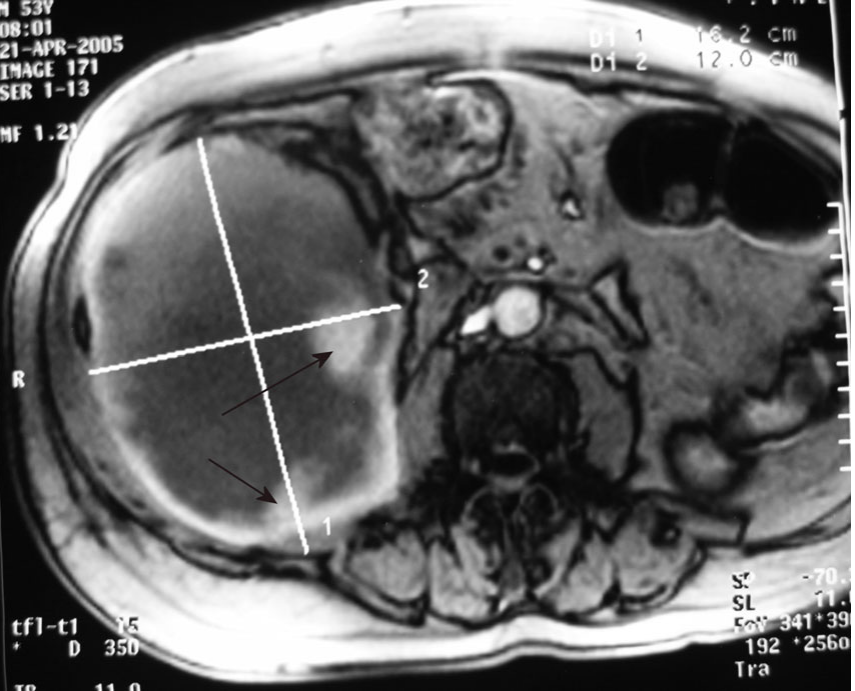
MRI – axial plan showing a large, mixed density mass in the right side of the abdomen suggestive of a large retroperitoneal hematoma, with areas of hyperdensity (arrows) indicating ongoing hemorrhage.

**Figure 3:**
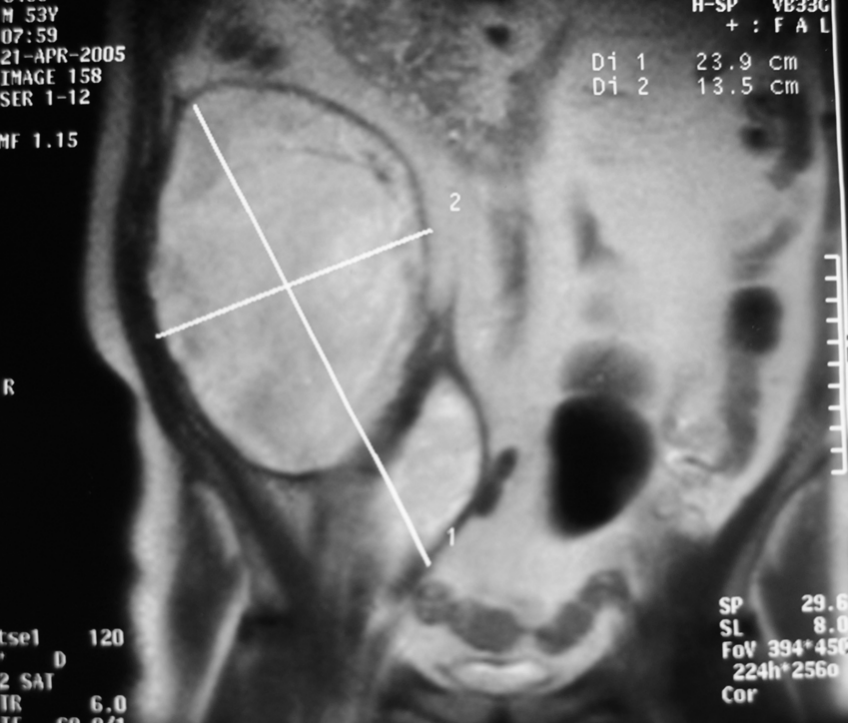
MRI – coronar plan.

Hematocrit level fell as did hemoglobin (Table 1). CT and magnetic resonance imaging (MRI) scan of the abdomen and the pelvis was obtained (Figures 1 and 2) and revealed extensive enlargement and heterogeneity of the right iliopsoas muscle as well as displacement of the right kidney. The high-attenuation component in the absence of intravenous contrast enhancement (Figure 3) is a finding that is usually consistent with the presence of a large retroperitoneal hematoma.

**Table 1:** Hematologic laboratory values

On admission		On fifth hospital day	
Variable	Value	Variable	Value

Hematocrit (%)	43	Hematocrit (%)	24.3
Hemoglobin (g/dl)	13.7	Hemoglobin (g/dl)	8.7
Mean corpuscular volume (μm^3^)	92	Platelet count (per mm^3^)	85,000
Erythrocyte sedimentation rate (mm/hour)	130	White-cell amount (per mm^3^)	14,900
White-cell amount (per mm^3^)	10,200	Prothrombin time (s)	16.1
*Differential count *(%)		Partial thromboplastin time (s)	63
Neutrophilis	64	D-dimer test (μg/l)	Negative^a^
Lymphocytes	27	Fibrinogen (mg/dl)	446^b^
Monocytes	7	Antithrombin III (mg/dl)	28^c^
Eosinophilis	1	Factor II (mg/dl)	14^d^
Basophilis	1	Factor V (mg/dl)	0.8^e^
Platelet count (per mm^3^)	265,000	Factor VII (mg/dl)	0.3^f^
		Factor X (mg/dl)	0.8^g^
		Prekallikrein (mg/dl)	5^h^

^a^Normal values less than 250 μg/l.

^b^Normal values in the range 200–400 mg/dl.

^c^Normal immunologic assay range 17–30 mg/dl.

^d^Normal values in the range 10–15 mg/dl.

^e^Normal values in the range 0.5–1 mg/dl.

^f^Normal value 0.2 mg/dl.

^g^Normal values in the range 0.6–0.8 mg/dl.

^h^Normal value 5 mg/dl.

Transfusion of six units of packed red cells and the administration of two units of fresh frozen plasma was followed by fluid overload. The patient was treated conservatively and his condition promptly stabilized after the restoration of normal blood coagulation; however, he remained in the hospital for 38 days. Three months later he had signs of partial lateral paresis of the right quadriceps muscle and thigh adductors and, at 1-year follow-up, the only findings were suggestive of the previous transient ischemic attack involving the carotid artery territory; he had recovered completely from the femoral neuropathy.

## Discussion

The large study of Sasson et al. [15] showed that patients who are receiving Heparin anticoagulation therapy, even in therapeutic doses, should be carefully monitored for the development of groin pain or leg weakness.

The most common symptoms are the acute onset, the severity and the persistence of the patient's pain in the lower abdominal quadrant, inguinal or lumbar region, and its radiation to the scrotum. Pain and paresthesia extend over the anterior, medial or lateral aspects of the lower extremities depending on the branches of the lumbar plexus that are involved. The most frequently involved nerve is the femoral nerve, the largest branch of the lumbar plexus which arises from the dorsal branches of L2, L3 and L4 ventral rami. It descends through the psoas major, emerging low on its lateral border and then passes between the psoas and iliacus, which makes the nerve vulnerable to traction injury from an underlying iliacus muscle hematoma [16,17], deep to the iliac fascia, passing behind the inguinal ligament into the thigh.

The diagnosis of atraumatic retroperitoneal hemorrhage remains challenging even when high-resolution MRI and CT imaging are used, because a large number of benign or malignant lesions can mimic this condition [18,19]. However, despite these limitations, MRI and CT imaging are superior to ultrasound and should be the preferred primary investigation [20-22].

The mainstay management currently consists of modification or cessation of anticoagulation therapy according to its clinical requirement, correction of the anticoagulation state, volume resuscitation and hemodynamic stabilization with adequate hematology and transfusion therapy and supportive measures [23]. Small hematomas with mild symptoms of neuropathy, without resultant obscuration, displacement or compression of normal retroperitoneal structures, without the need for multiple transfusions and without signs of infection may be treated conservatively.

On the other hand the effectiveness and safety of surgical intervention and evacuation of the hematoma should be considered as a potential strategy in uncontrollable hemodynamic collapse or when the nerve involved in the decompression might be effective in that the direct pressure and pressure-induced ischemic effects are reversible [24,25]. The latter is limited by the inability to localize or control the bleeding vessel and the risk of worsening the bleeding by releasing the tamponade [26].

## Conclusion

The rarity of this possible complication of the intravenous use of Heparin in patients with INR < 4.5 means that it remains a challenge for surgeons. We strongly suggest that, according to our experience, daily measurement of INR and activated partial thromboplastin time (aPTT) in patient's receiving Heparin intravenously as an anticoagulation agent is of great importance. In deep vein thrombosis or acute myocardial infarction, the usual protocol requires injection of Heparin monitored by the prothrombin time, aPTT or both followed by long-term therapy with oral anticoagulants. As the half-life of Heparin is 3 hours, we suggest that aPTT to be measured 3 hours after Heparin administration or 1 hour before the next dose.

Some of the most important factors for the diagnosis are acute onset of pain, a dramatic change in the patient's clinical status and high clinical suspicion. CT and MRI remain the most powerful diagnostic tools. The complex challenge for the surgeon is the choice of clinical pathway in the management of this rare entity and this choices should only be made after taking two key points into consideration: (i) the patient's general condition; (ii) in the presence of permanent femoral or sciatic neuropathy due to a compression syndrome, hemodynamically unstable patients should be managed with an emergency laparotomy.

## Competing interests

The authors declare that they have no competing interests.

## Authors' contributions

SID participated in the sequence alignment, in the design of the case report and drafted the manuscript. AB participated in the design of the case report. DP participated in the design of the case report and coordination. PP participated in the design of the study. All authors read and approved the final manuscript.

## Consent

Written informed consent was obtained from the patient for publication of this case report and accompanying images. A copy of the written consent is available for review by the Editor-in-Chief of this journal.
